# Ichthyosiform Lichen Planus Pigmentosus in a 19-Year-Old Male Patient: Case Report

**DOI:** 10.2196/50429

**Published:** 2024-04-19

**Authors:** Audi Sugiharto, Julius Gatmaitan, Johannes Dayrit

**Affiliations:** 1 Department of Dermatology Research Institute for Tropical Medicine Muntinlupa City Philippines; 2 Skines Aesthetic and Laser Center Bulacan Philippines; 3 Department of Internal Medicine De La Salle University Medical Center Dasmariňas City Philippines

**Keywords:** pigmentary disorder, lichen planus pigmentosus, ichthyosiform, asymptomatic, pigmentation, sun exposed, hypersensitivity, diffuse, hyperpigmentation, clinical, skin, dermatologist, dermatology, Filipino, Pacific Island, sun, sunburn

## Abstract

Lichen planus pigmentosus (LPP) is a condition characterized by persistent and asymptomatic brownish-black–to-blue or purple-gray pigmentation, predominantly in the face and sun-exposed areas, commonly in dark-skinned individuals. Several clinical variants of LPP have been reported. However, the ichthyosiform type of LPP has not been reported. We present a 19-year-old male patient who presented with a 7-year history of asymptomatic grayish macules; patches with fine scales on the face, trunk, and upper extremities; and grayish plaques with thick “ichthyosiform” scales on the lower extremities. The diagnosis of LPP was proven by histopathological findings on both the macular and ichthyosiform plaques. Cluster differentiation (CD) 68 stain highlights the same density of pigment-laden macrophages in both the gray macule and the ichthyosiform plaque. The cause of LPP is unknown. Transcription factor anomalies may play a role in increased keratinization of lichen planus lesions. It can be assumed that the mechanism of the altered distribution of keratinization may occur on the ichthyosiform lesions in this patient. The terminology “ichthyosiform lichen planus pigmentosus” is hereby proposed to be added to the clinical variants of LPP.

## Introduction

Lichen planus (LP) is an inflammatory disorder affecting skin, mucous membranes, nails, and hair with prototypic “lichenoid” papules. LP has a worldwide distribution with incidence varying from 0.22% to 1% depending on the geographic location [1]. LP can involve the skin or mucous membranes (oral, vulvovaginal, esophageal, laryngeal, and conjunctival mucosa). This condition has different variants based on the morphology of the lesions and the site of involvement [2].

Subtypes based on the configuration or morphology of the lesions include the following: popular (classic), hypertrophic, vesiculobullous, actinic, annular, atrophic, linear, follicular, and LP pigmentosus (LPP) [2]. LPP is a variant of LP characterized by hyperpigmented macules in sun-exposed areas and flexures of dark-skinned individuals [3]. The pigmentation is dermal and occurs without any clinical evidence of inflammation [3].

The cause of LPP is unknown. The diffuse and symmetric classical type, linear unilateral hyperpigmentation in the extremities (Blaschkoid), and segmental patterns on the trunk have been documented. Reticular, blotchy, perifollicular, annular, and gyrate patterns are also encountered [4]. Another rare variant of LPP, that is, LPP inversus located on skinfold areas, has also been reported [5]. However, ichthyosiform variant of LPP has not been reported.

## Case Report

**Figure 1:**
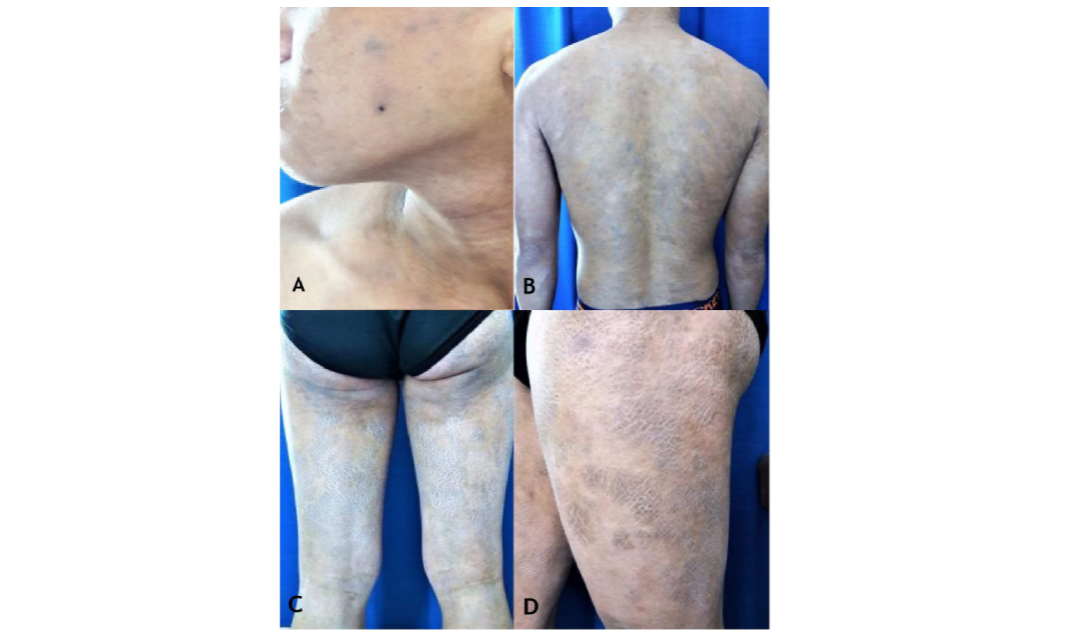
Clinical findings: “asymptomatic grayish macules; patches with fine scales on the face (A), trunk, and upper extremities (B); and grayish plaques with thick “ichthyosiform” scales on the lower extremities (C and D).

A 19-year-old Filipino male patient presented with a 7-year history of asymptomatic grayish macules; patches with fine scales on the face, trunk, and upper extremities (Figure 1A and 1B); and grayish plaques with “ichthyosiform” scales on the lower extremities (Figure 1C and 1D).

**Figure 2:**
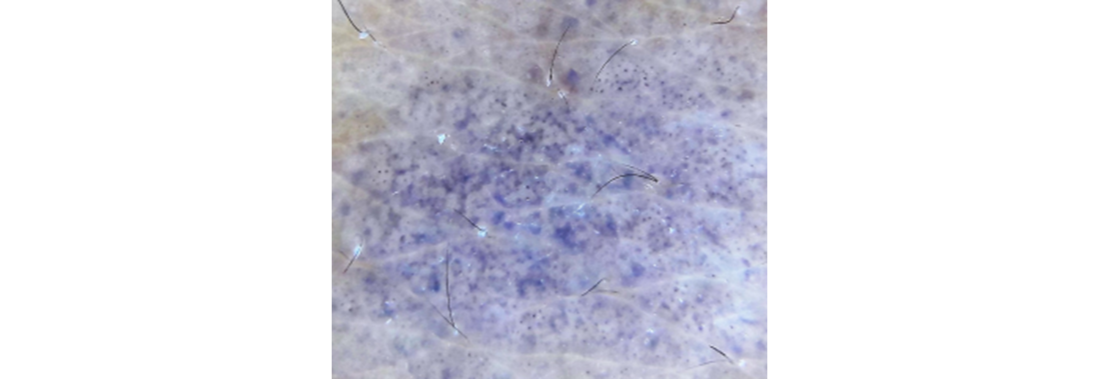
Dermoscopy shows black dots and globules in a “hem-like” and reticular pattern (Dermlite DL3 polarized dermoscopy).

We used a manual polarized light device (Dermlite DL3x10, 3Gen). The dermoscopic finding shows dots and globules in a “hem-like” and reticular pattern, which spares the follicular opening (Figure 2).

**Figure 3:**
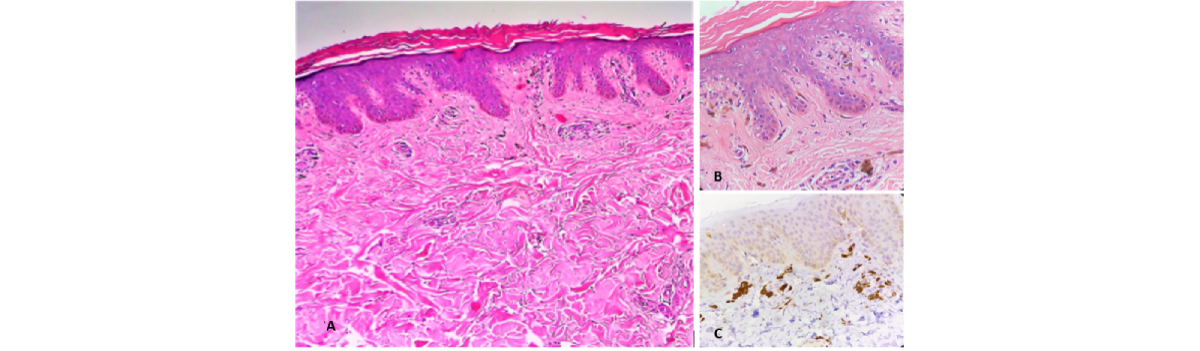
Histopathology of the ichthyosiform gray plaque shows (A) epidermal hyperkeratosis and hypergranulosis with acanthosis and multifocal areas of vacuolar alteration of the basal cell layer (H&E×100). (B) Numerous pigment-laden macrophages and mild perivascular inflammatory infiltrate of lymphocytes can be appreciated from the dermis (H&E×400). (C) CD68 immunostaining highlights the large pigment-laden macrophages on the biopsy of the ichthyosiform plaque (CD68×400). CD: cluster differentiation; H&E: hematoxylin and eosin.

A 4-mm skin punch biopsy was performed on 2 separate sites (the macule and the ichthyosiform plaque). Histopathology of the ichthyosiform plaque revealed hyperkeratosis and hypergranulosis of the stratum corneum with acanthosis and multifocal areas of vacuolar alteration of the basal cell layer. Histopathology results of both specimens presented with numerous pigment-laden macrophages and mild perivascular inflammatory infiltrate of lymphocytes in the dermis (Figure 3A and 3B). Cluster differentiation (CD) 68 immunostaining highlights the same density of pigment-laden macrophages in both the gray macule and the ichthyosiform plaque (Figure 3C). Definitive diagnosis of LPP was proven by histopathological findings on both the macule and ichthyosiform plaque.

Direct immunofluorescence of the 4-mm skin punch biopsy from the lesional area of the right arm revealed negative results. Serial sections showed no immunofluorescence for immunoglobulin (Ig) A, IgG, IgM, and complement C3 and fibrinogen for epidermis, basement membrane zone, and vascular areas.

## Discussion

LPP is a rare variant of LP that is seen in individuals with darker pigmented skin [3,4]. The etiology of this condition still remains unknown, but a number of agents have been reported to act as predisposing factors [4]. In 2014, a global consensus statement on acquired macular pigmentation of uncertain etiology concluded that LPP is unlikely to be caused by sociocultural practices or particular dietary ingredients [6].

The occurrence of this condition primarily in sun-exposed areas in numerous patients has led to the proposition that sunlight may be a principal etiological agent [4]. Clinical manifestations of LPP lesions can be found in sun-exposed areas as well as non–sun-exposed areas [6]. For the sites of predilection, LPP involves the head and neck region in most cases followed by the involvement of flexural area, particularly the axillae [6]. Although rare, the involvement of sun-protected areas such as trunk and thigh has also been reported [3,7], similar to our patient.

A number of other variants such as localized LPP (on thigh), segmental LPP, LPP inversus at the skinfold area, linear LPP, LPP in zosteriform distribution, LPP along lines of Blaschko, and LPP of oral mucosa have been reported [4]. Reticular, blotchy, perifollicular, annular, and gyrate patterns were also encountered [4]. LPP with an ichthyosiform pattern similar to our patient has not been reported.

LPP manifests as pigmentation of insidious onset without any features of inflammation or preceding raised lesions. It is typically asymptomatic and may occasionally be accompanied by mild pruritus. The course is variable, with some cases showing spontaneous resolution within weeks to months. It may be persistent over the years in many [3].

Dermoscopy of LPP lesions revealed pigmentation in different nonspecific patterns. These dotted patterns described as fine or coarse blue-gray dots correspond to melanophages in the dermis. Mixed patterns correspond to lesions showing both epidermal and dermal components. In our case, dermoscopy shows dots and globules in a “hem-like” and reticular pattern similar to the findings of Mathews et al [3].

Histologic features of LPP and LP are similar [3,8]. LPP is characterized by interface dermatitis with dense lichenoid reaction in the dermis with pigmentary incontinence and the presence of melanophages [3,7]. The inflammatory phase is characterized by a dense band of lymphohistiocytic inflammatory infiltrate in the upper dermis with prominent basal vacuolar degeneration. Some melanin incontinence is seen with scattered dermal melanophages [3]. These findings are compatible with our patient’s histopathological findings that are more compatible with LPP. In classic LP, additional findings of wedge-shaped hypergranulosis, saw-toothing of the rete ridges, colloid bodies, and a more prominent lichenoid inflammatory infiltrate of lymphocytes are further observed [9].

LPP is considered as a variant of LP [3]. It has a well-described association with classical lesions of LP [3,8]. The pathogenesis of LPP is not yet widely known but postulated to be secondary to type IV hypersensitivity reaction or T-lymphocyte–mediated cytotoxic activity against basal keratinocytes [3,5]. It has been proposed that barrier impairment may be a preceding event in the pathogenesis of LP, or it may occur as a secondary effect resulting from a disturbance in keratinocyte differentiation. A number of studies also revealed that certain transcription factors in LP increased expression of the differentiation-related genes involucrin, filaggrin, and loricrin, which play a role in the keratinization of cutaneous LP lesions [10]. Altered distribution of filaggrin was also observed in patients with LP in other cited literatures [11]. Taking into consideration all the possible pathogenesis of the condition, it is safe to assume that the mechanism of the altered distribution of keratinization in ichthyosiform LPP is similar to what we found in this patient.

The complex relationship between keratinization abnormalities and cutaneous inflammatory illnesses is highlighted by the appearance of ichthyosiform plaques in LPP lesions. Rigid clinicopathological connection and increased dermatologist awareness of this rare clinical presentation are necessary for an accurate diagnosis. In conclusion, the terminology “ichthyosiform lichen planus pigmentosus” is hereby proposed to be added to the clinical variants of LPP. A case series of ichthyosiform LPP is further recommended to confirm this new terminology.

## Abbreviations

CD: cluster differentiation
Ig: immunoglobulin
LP: lichen planus
LPP: lichen planus pigmentosus
